# Atomic‐Scale Study of Cation Ordering in Potassium Tungsten Bronze Nanosheets

**DOI:** 10.1002/advs.201600537

**Published:** 2017-04-26

**Authors:** Luying Li, Fan Jiang, Fanfan Tu, Shuangfeng Jia, Yihua Gao, Jianbo Wang

**Affiliations:** ^1^ Center for Nanoscale Characterization and Devices Wuhan National Laboratory for Optoelectronics School of Physics Huazhong University of Science and Technology Wuhan 430074 China; ^2^ School of Physics and Technology Center for Electron Microscopy and MOE Key Laboratory of Artificial Micro‐ and Nanostructures Wuhan University Wuhan 430072 China

**Keywords:** cation vacancy ordering, hierarchical nanostructures, potassium tungsten bronze, probe‐corrected HAADF imaging

## Abstract

It has long been accepted that the formation of superlattices in hexagonal‐based potassium tungsten bronzes is attributed to K vacancies only, together with small displacements of W cations. Here, the superlattices within potassium tungsten bronze nanosheets both structurally and spectroscopically at atomic resolution using comprehensive transmission electron microscopy techniques are studied. The multidimensional chemical analyses are realized by energy‐dispersive X‐ray spectroscopy, electron energy‐loss spectroscopy, and X‐ray photoelectron spectroscopy, the atomic‐scale structures are characterized using aberration‐corrected scanning transmission electron microscopy with high‐angle annular‐dark‐field detector. The observed superstructures are mainly attributed to small amount of W vacancies within single atomic layer, which would recover to more uniform distributions of W vacancies with lower concentrations at higher temperature. The band regions of different orientation from the matrix tend to regulate the superstructures to be pinned along the same direction, forming domains of highly ordered structures. The characterization of cation ordering and recovery processes of nanostructures from chemical and structural point of view at atomic resolution enables rational design of optoelectronic devices with controlled physical properties.

## 1 Introduction

Potassium tungsten bronze is a group of nonstoichiometric compounds with remarkable electrical and chemical properties, which could be synthesized into nanowires,^1^, ^2^, ^3^ nanobundles,^4^ and nanosheets^5^, ^6^, ^7^ depending on the growth temperatures. The applications include electron field emission,^3^ high‐sensitive gas sensors,^7^ solar filter,^8^ to name a few. The tungsten bronzes have the general formula M*_x_*WO_3_, where M denotes specific alkali metal, and their crystal structures are closely related to the size and amount of the alkali metal, which normally include four structural types: perovskite, tetragonal I, hexagonal tungsten bronze (HTB), and intergrowth tungsten bronze.^9^

The hexagonal tungsten bronzes with 0.19 ≤ *x* ≤ 0.33 are formed by WO_6_ octahedra linked by corners. The potassium atoms are located in the hexagonal tunnels, and the value of *x* equals 0.33 while all available tunnel positions are occupied. With decreasing values of *x*, vacant positions occur in the original potassium lattice. The hexagonal K*_x_*WO_3_ structure with lattice parameters *a* = 7.37 Å and *c* = 7.56 Å complies with space group *P*6_3_/mcm and JCPDS 49‐0541.^10^, ^11^

Back in 1976, Goodman and Mclean reported transmission electron micrographs of ordering in K*_x_*WO_3 +_ *_y_* (x ≈ 0.27 ± 0.02) as faint parallel lines. The signal‐to‐noise ratio of the lattice fringes was enhanced by deliberately adjusting the objective stigmator. The observed superlattices were attributed to the ordering of potassium vacancies based on charge‐density calculations.^12^ It is claimed that a monoclinic supercell of potassium tungstate is possible with *a*_M_ = 4 *a*_H_, *b*_M_ = 2 *b*_H_, *c*_M_ = *c*_H_ (γ = 120°) (M stands for monoclinic, H stands for hexagonal structures), where the quadrupling of *a*_H_ is resulted from ordering of potassium vacancies in the tunnels, and the doubling of *b*_H_ is caused by small displacements of tungsten atoms within WO_6_ octahedra.^13^

Our previous studies also reported ordered monoclinic superstructures in hexagonal‐based potassium tungsten bronze nanosheets based on the experiment and simulation of selected area electron diffraction (SAED). The satellite superstructure peaks were resulted from ordered arrangements of K vacancies and the coexistence of 120° rotation twinning variants.^5^ The threefold twinning variants distributed in the nanosheets were thought to be formed by oriented attachment of neighboring nanowires.^6^

While in most cases the refinement of superstructures is achieved using X‐ray, neutron or electron diffraction techniques that provide average structural information in the reciprocal space,^14^, ^15^, ^16^, ^17^ direct observations of superstructures at atomic resolution are possible via the negative spherical aberration imaging technique,^18^ and the combined application of high‐angle annular‐dark‐field (HAADF) electron microscopy and core‐level spectroscopy.^19^

Here, direct observation of superstructures in hexagonal‐based potassium tungsten bronze nanosheets is realized both spectroscopically and structurally at subangstrom resolution using scanning transmission electron microscope (STEM) with a probe corrector, an HAADF detector and a ChemiSTEM attachment of FEI. From cross‐sectional view, the nanosheets show superstructures of long‐periodic cation ordering, and the temperature dependence of the recovery from cation ordering is also studied.

## 2 Results and Discussion

**Figure 1:**
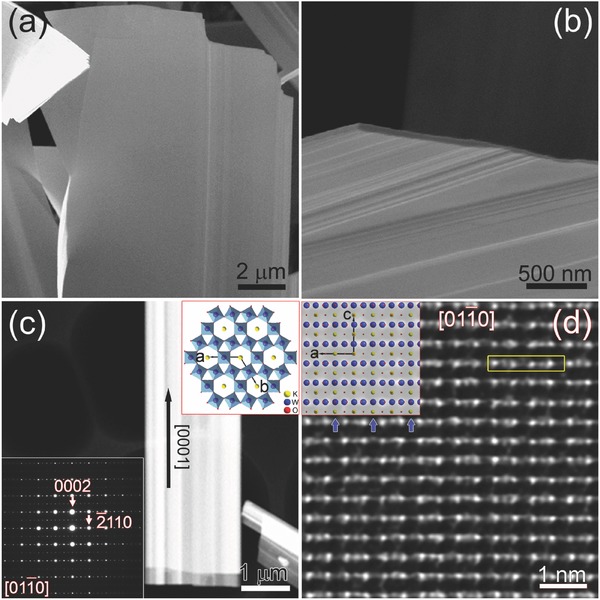
a) Plan view and b) cross‐sectional view SEM images of individual K*_x_*WO_3_ nanosheet, respectively. c) HAADF image of a piece of K*_x_*WO_3_ nanosheet lying on the holy carbon film with growth direction along [0001]. The upper right inset is the atomic model of hexagonal K*_x_*WO_3_ projected along *c* axis. The lower left inset is SAED pattern of the selected nanosheet projected along $\left[01\bar{1}0\right]$. d) High‐resolution HAADF image of K*_x_*WO_3_ nanosheet projected along $\left[01\bar{1}0\right]$. The inset is corresponding atomic model projected along the same direction. The blue arrows indicate tungsten columns of much lower intensities appearing periodically as dark lines.

**Figure** **1**a,b is plan view and cross‐sectional view of potassium tungsten bronze nanosheets using scanning electron microscope (SEM). The nanosheets show vast 2D surface areas with thicknesses ranging from 15 to 100 nm. It is found that the nanosheets have rough surfaces with step‐like outlines. The HAADF–STEM image intensity directly scales with the atomic number Z giving Z contrast.^20^ In previous studies, the aberration‐corrected HAADF–STEM technique has been applied to characterization of local faulted structures^21^ as well as analysis of related structure–property relations^22^ at atomic resolution. Figure 1c is HAADF–STEM image of a small piece of K_x_WO_3_ nanosheet with growth direction along [0001]. Parallel dark contrasts appear along the *c* axis, which can also be attributed to the rough surfaces and uneven projected thicknesses. The upper right inset of Figure 1c is the atomic model of hexagonal K*_x_*WO_3_ projected along *c* axis, which shows the corner‐sharing WO_6_ octahedra and the distribution of potassium atoms in the hexagonal tunnels.

The lower left inset is the corresponding SAED pattern projected along $\left[01\bar{1}0\right]$ with the nanosheet laid on the holy carbon film. While the major diffraction spots could be attributed to the hexagonal tungsten bronzes structure (Figure S1a, Supporting Information), the fourfold satellite spots indicate the existence of an ordered superstructure. The powder X‐ray diffraction (XRD) spectrum of potassium tungsten bronze nanosheets is shown in Figure S1b (Supporting Information). The major peaks could be indexed according to the hexagonal K*_x_*WO_3_ structure, while the 0002_H_ and 0004_H_ reflections are the strongest. There are also reflections from WO_3_ and W as impurities. Besides that, satellite superstructure peaks exist as indicated by black arrows, which could not be indexed as either hexagonal K*_x_*WO_3_ structure or other possible impurities. They were explained as monoclinic phases with 120° rotation twinning variants in previous studies.^5^, ^6^

Figure 1d is high‐resolution HAADF–STEM image of the above selected nanosheet projected along $\left[01\bar{1}0\right]$ direction. The corresponding atomic model is overlapped on the upper left corner based on the crystallographic data of K_0.20_WO_3_ with slight adjustment of the tungsten atomic positions to allow better coincidence with the experimental results.^11^ It is found that the white spots in Figure 1d correspond to tungsten atomic columns. The potassium atoms are expected to be located between tungsten layers. However, the contrasts of potassium columns are sparsely shown in the experimental image, which might be due to much lower atomic number of potassium as compared to tungsten (K:19, W:74), and the possible existence of potassium vacancies. Tungsten columns of much lower intensities appear periodically as dark lines in the experimental image, as labeled by blue arrows. The comparison with the hexagonal tungsten bronze atomic model reveals that the uneven contrasts of tungsten columns might be due to different densities of tungsten atoms along $\left[01\bar{1}0\right]$ axis (Figure S1c,d, Supporting Information), with no obvious signature of superstructures. Thus, the plan view scheme is not the proper way to investigate the superstructures.

**Figure 2:**
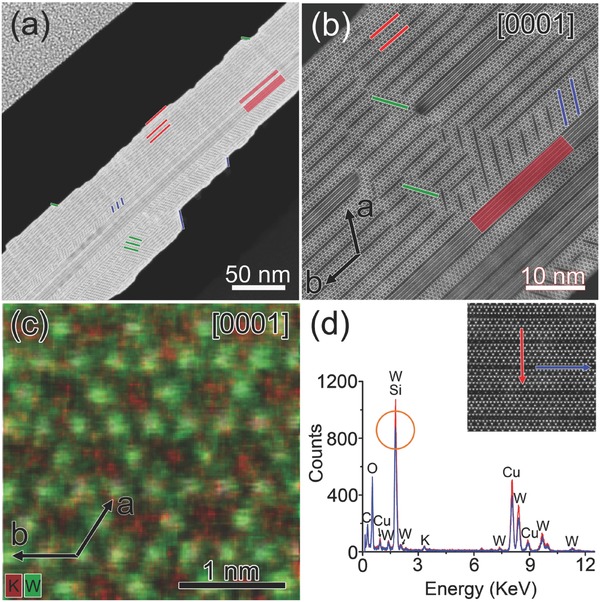
a) HAADF image of cross‐sectional view of individual nanosheet. b) High‐resolution HAADF image of the same nanosheet, showing the structural features at higher magnification. c) Red–blue false‐color image by combining the K and W elemental maps at atomic resolution. The semitransparent HAADF image of the same region is overlapped to allow direct structural and spectroscopical comparison. d) Integrated EDS spectra along the regions labeled by red and blue arrows in the upper right HAADF image.

In order to obtain direct experimental proof of the superstructures in K*_x_*WO_3_ nanosheet on an atomic basis, cross‐sectional sample for individual nanosheet is prepared by focused ion beam (FIB). **Figure** **2**a is HAADF–STEM image of K*_x_*WO_3_ nanosheet observed end‐on. Figure 2b is corresponding higher magnification HAADF–STEM image, which better presents the detailed structural features. The cross section of K*_x_*WO_3_ nanosheet is a thin piece, with parallel dark lines of equal spacing mostly following the direction of the edges, as labeled by the red lines. Parallel dark lines in the other two directions also appear following the edges labeled by the green and blue lines, which leads to the step‐like outlines of the surfaces. The dark lines along the red, blue and green lines conform to threefold rotational symmetry, which reflects the same symmetry of the lattices along ***c*** axis. Besides that, there are parallel wide bands penetrating through the center of the piece, as shown by the red rectangle. The corresponding atomic structures as well as their influence on the superstructure formation within the K*_x_*WO_3_ nanosheet would be discussed later.

Determination of chemical information at atomic resolution holds fundamental importance for unraveling the correlation between material structures and related physical properties. Yet, the atomic chemical analyses were traditionally achieved using aberration‐corrected STEM in combination with electron energy‐loss spectroscopy (EELS)^23^, ^24^, ^25^, ^26^ rather than energy‐dispersive spectroscopy (EDS) since the former has much higher energy resolution as well as spatial resolution. With the emergence of liquid‐nitrogen‐free silicon drift detector with optimized collection solid angle, chemical mapping at atomic resolution by aberration‐corrected STEM–EDS becomes possible.^27^ In the current study, the edge energy of tungsten is 1809 eV, which would be extremely difficult to detect by EELS for samples of moderate thicknesses, and aberration‐corrected STEM–EDS would be the better choice.

Figure 2c is 2D STEM–EDS elemental map of the cross‐sectional sample projected along [0001] at atomic resolution. The red‐green false‐color image combines the K and W elemental maps, and the semitransparent HAADF–STEM image of the same region is overlapped to allow direct structural and spectroscopical comparison. It is found that the green contrasts in the elemental map coincide with the white spots in the semitransparent HAADF–STEM image, indicating that the white spots should correspond to tungsten columns as expected. The red contrasts in the elemental map are located right within the hexagonal tunnels in the HAADF–STEM image, which are signatures of potassium columns.

Moreover, EDS spectra at individual pixels are integrated along the regions labeled by red and blue arrows in the upper right HAADF–STEM image in Figure 2d. The two regions are of the same thickness and length, while the red arrow mostly covers the background hexagonal lattices, the blue arrow solely goes through the dark‐line region. The resulting EDS spectra indicate the existence of K, W, O as expected. The C and Cu signals should come from surface contaminants and copper grid as supporter, respectively. Careful examination of the integrated peak intensities in the EDS spectra reveals that all of the W peaks are stronger in the region of the red arrow than that of the blue arrow, suggesting the existence of W deficiencies within the dark‐line region.

Some may argue that the observed dark lines could also be attributed to the presence of lighter elements as impurities on the supposed W positions. There is no deliberate introduction of dopants during material growth, and the EDS spectra randomly collected over large areas in the specimen show no extra elemental signals except for C and Cu. Based on the extensive EDS studies, the value of *x* in K*_x_*WO_3_ nanosheet is calculated according to the atomic percentage of K and W. It is found that *x* varies from area to area, mostly in the range of 0.18–0.22, which might be due to uneven distribution of K ions within the hexagonal tunnels, or the inaccuracy induced by the EDS technique, which is not accurate enough in quantitative analysis of the chemical information without calibration with standard samples. On the other hand, it is reasonable to have variable K contents since the K ions are moveable within the hexagonal tunnels. Meanwhile, EELS spectra are also collected over characteristic energy ranges (see Figure S2 in the Supporting Information). The K–L_2,3_ edges (294, 296 eV) and O–K edge (532 eV) are clearly shown, while the C–K edge at 284 eV and Cu–L_2,3_ edges at 931 and 951 eV are absent, indicating that the K*_x_*WO_3_ nanosheet does not include these two elements.

One needs to be cautious about the W peak close to 2 KeV, which is circled out in Figure 2d. Since the W peaks (*M*_α_ = 1.78 KeV, *M*_β_ = 1.84 KeV) and the Si peaks (*K*_α_ = 1.74 KeV, *K*_β_ = 1.83 KeV) are very close here. It is necessary to determine whether Si is present through separate techniques since Si has no other characteristic peaks in the EDS spectrum. X‐ray photoelectron spectroscopy (XPS) is an important technique for chemical analysis of material surfaces. It measures the binding energies of certain elements based on photoelectric effect. The K*_x_*WO_3_ nanosheets on W substrate in their original state after growth are examined by XPS (see Figure S3 in the Supporting Information), the featured peaks for O 1s (531.104 eV) and K 2p (293.354 eV) are obvious, and the peak for Si 2p (101.554 eV) is absent, which proves the absence of Si in the nanosheets. The products are scratched off the W substrate and loaded on a Si substrate, the corresponding XPS spectra shows obvious W 4f (31.104 eV) signals. Thus, the observed dark‐line superstructures should be attributed to deficient W columns based on comprehensive spectroscopic analyses.

**Figure 3:**
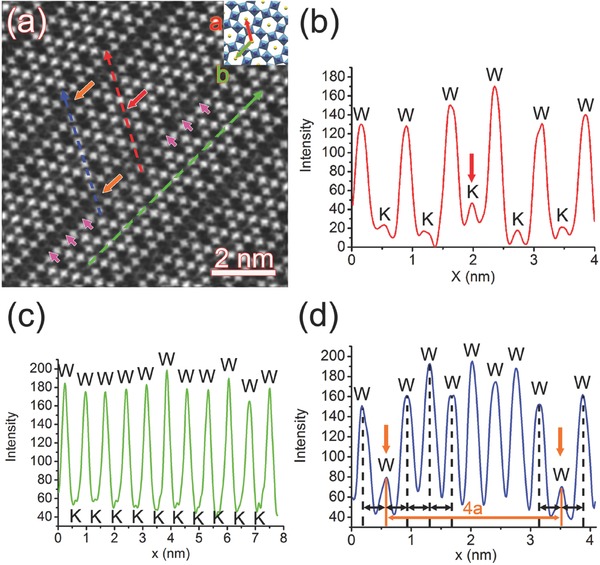
a) High‐resolution HAADF image of K*_x_*WO_3_ nanosheet projected along *c* axis, including parallel dark lines of equal spacing. The inset is the atomic model of hexagonal K*_x_*WO_3_ projected along *c* axis overlapped on the experimental image. The red, green, and navy dashed arrows indicate locations of intensity line profiles, and the results are presented in (b), (c), and (d), respectively. The corresponding atom types are labeled on the top of each intensity peaks.

The related structural analysis is carried out applying probe‐corrected high‐resolution HAADF–STEM imaging of a region including parallel dark lines of equal spacing in K*_x_*WO_3_ nanosheet projected along *c* axis at atomic resolution, as shown in **Figure** **3**a, where single atomic columns are clearly resolved. The inset is the corresponding atomic model overlapped to indicate the locations of specific atom types: the tungsten columns form hexagonal matrices, and the potassium columns are supposed to reside in the tunnels surrounded by those hexagons. It is found that the dark‐line regions are composed of both tungsten and potassium column positions.

To analyze the atomic distributions of the superstructures, intensity line profiles along specific directions, as labeled by red, green and blue dashed arrows in Figure 3a, are presented in Figure 3b–d, respectively. The red dashed arrow, which is along *a* axis, goes through tungsten and potassium columns alternately, and the corresponding peaks are labeled as “W” and “K” in Figure 3b. The intensities for “W” peaks are not exactly the same with the maximum discrepancy of 20.2% $\left(\frac{I_{\max}-I_{\mathrm{mean}}}{I_{\mathrm{mean}}}\times 100\%\right)$, indicating that the sample surfaces are not atomically flat. Moreover, the intensities of the K sites right within the dark lines are slightly higher than those of the adjacent K sites, while the “K” peak in the center has much higher intensity than the others with maximum discrepancy of 84.1%, which is obviously higher than what is expected from the noise or local thickness variations. Thus, the potassium column labeled by a red arrow in Figure 3a should have higher density than the other four columns selected by the red dashed arrow. The same abrupt increase of “K” column density happens periodically every four “K” columns. Although the accurate interpretation of atomic structures could be disturbed by the overall poor signal‐to‐noise ratio or the tails of the electron probe, the latter would lead to intensity transfer from one atomic column to neighboring atomic columns,^28^ all of the atomic columns would be affected uniformly with no abrupt intensity variation of specific columns as in our case.

Figure 3c is intensity line profile along −*b* direction, which also goes through alternating tungsten and potassium columns. It seems that the peak intensities for both tungsten and potassium columns are comparatively constant, with maximum discrepancies of 10.1% for “W” and 13.6% for “K” peaks, respectively. Figure 3d is intensity line profile also along *a* axis, which only goes through tungsten columns. It is apparent that the “W” peaks labeled by orange arrows have much lower intensities than the others do. The locations of the tungsten columns with lower intensities correspond to the dark lines labeled by orange arrows in Figure 3a, which are four‐periods apart. Thus, it could be concluded that both tungsten and potassium columns participate in the formation of superstructures with period of 4a along *a* axis, while no sign of long‐periodic ordering exists along *b* axis.

Parallel superstructures in the intergrowth tungsten bronzes are reported,^29^, ^30^ which also show parallel dark lines and somewhat resemble the current case. Those reported structures involve oxygen octahedra that can form distorted perovskite slabs. In the current study, the crystal structure right at those dark lines has the same structural symmetry as the surrounding regions without any abrupt structural change, which should be different from the case of intergrowth tungsten bronzes.

What really happens to the crystal structures at the dark‐line regions? One interpretation would be the existence of W vacancies. Not fully occupied K positions are quite common in hexagonal‐based potassium tungsten bronzes, which would not influence the framework of the HTB structure but only the oxidation state of W. The existence of small amount of W vacancies, on the other hand, would lead to local structural distortions with displacements of W atoms from the ideal sites, which would decrease the local HAADF intensity based on integration along the atomic columns in the direction of the electron beam. Close inspection of the atomic structures right at the dark lines reveals no detectable distortions of the W—W bonds, as indicated by the black double arrows of the same length in Figure 3d. Since no observable collapse of the octahedral framework is observable at the dark‐line region, the appearance of large number of cation vacancies within the polyhedrons inside the dark lines is not possible in the current case.

The comprehensive spectroscopical analyses exclude the possibility of substitution of W positions for impurities of lighter elements. H is difficult to detect by EDS or EELS, and water might be formed by heating. While the structural study of hydrated samples of Na*_x_*WO_3 +_ *_x_*_/2_·*y*H_2_O (*x* ≈ 0.17, *y* ≈ 0.23) reveals that the water molecules are found in the hexagonal cavity, the octahedral framework is not disturbed with no influence on the local HAADF intensity.^9^ Another possibility would be an intact octahedral framework with varying W and K occupations at the boundaries, which would lead to decreased average atomic number (density) and lower HAADF–STEM intensities in the original W column positions. However, the antisite occupations of W and K atoms are highly unexpected from size and charge considerations, which have not been reported in previous studies.

**Figure 4:**
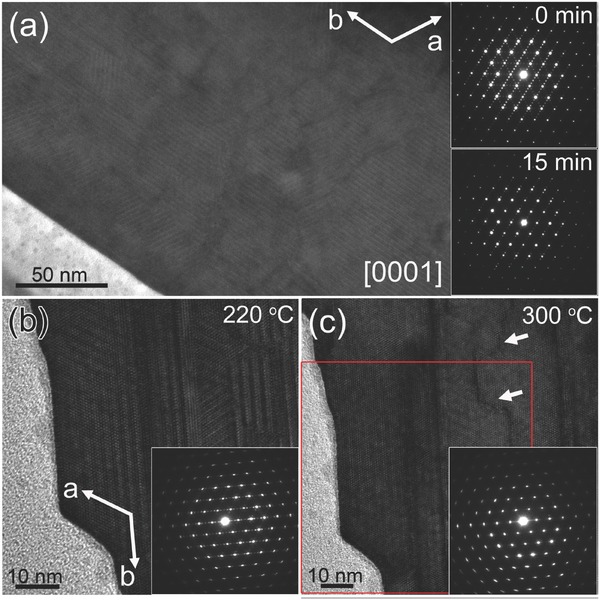
a) TEM image of cross‐sectional view of specific nanosheet showing parallel dark lines of 120° rotational symmetry before electron beam irradiation. The insets are corresponding SAED patterns before and after 15 min of irradiation by parallel electron beams. b) HRTEM image of cross‐sectional view of another nanosheet including dark‐line superstructures observed at 220 °C. The inset is corresponding SAED pattern showing satellite spots. c) HRTEM image of the same nanosheet observed at 300 °C. The region framed in a red box corresponds to the same region in (b). The dark‐line superstructures almost disappear along with the satellite spots in the corresponding SAED pattern shown in the lower right inset. Irregular Crack lines start to form at 300 °C, as indicated by white arrows.

It is found that under continuous irradiation of focused electron beam with acceleration voltage of 300 kV, the original parallel dark lines become gradually blurred, and finally vanished leaving the background lattices with hexagonal periodicity at atomic resolution (see Movie S1 in the Supporting Information). To further investigate the mechanism of the recovery process, specific area of the cross‐sectional sample is irradiated by parallel electrons with acceleration voltage of 200 kV for 15 min, and the corresponding SAED patterns are obtained before and afterward, as shown in **Figure** **4**a. The fourfold satellite spots still exist, although they are not as clear as before due to electron beam damage to the specimen. In situ heating experiment is also carried out to study the temperature dependence of the superstructures. When the temperature is increased to 220 °C from room temperature, the crystal structure is almost unchanged, as presented in Figure 4b. However, by increasing the temperature to 300 °C, the superstructures quickly disappear in a few seconds, and the satellite spots in the corresponding SAED pattern disappear as well (Figure 4c). Moreover, when the temperature is increased from 220 to 300 °C, the *x* value (K content as compared to W) does not show any abrupt change ($x_{220\;{}^{\circ}C}=0.19,x_{300\;{}^{\circ}C}=0.20$), indicating no obvious cation loss during the process of reorder. However, when the temperature is kept at 300 °C for more than 30 min, the *x* value is apparently decreased to 0.12, which could be attributed to K volatilization under continuous heating at high temperature. Thus, it is quite possible that the recovery of the superstructures is mainly induced by increased temperature rather than electron beam radiation.

The K*_x_*WO_3_ nanorods are also synthesized at lower temperatures (growth temperature of 450 °C for 10 h), which show completely different features of the superstructures (see Figure S4 in the Supporting Information). No wide‐band region is found in nanorods, and the dark lines show no preference of orientation. When the dark lines from different directions meet together, the original periodicity would be disturbed, introducing structural disorders at the intersecting regions, which seem to be much more sensitive to electron beam irradiation with resultant holes at the center as labeled by orange circles. This complete structural disorder has never been found in the K*_x_*WO_3_ nanosheets. Based on the above observations, it seems to be very possible that small amount of W vacancies are present in the dark lines of single atomic‐layer thick, which would explain the lower EDS counts of W in the dark‐line region as compared to the hexagonal matrix, and the lower local HAADF intensity in the dark‐line region. Through annealing or heating, the surrounding W ions are inclined to move to the W vacancy sites, forming structures with more evenly distributed W ions. During the annealing process, no extra vacant cation site is formed but just movement of cations among different cation sites. Thus, no excess cation is needed to fill the extra vacant cation sites.

**Figure 5:**
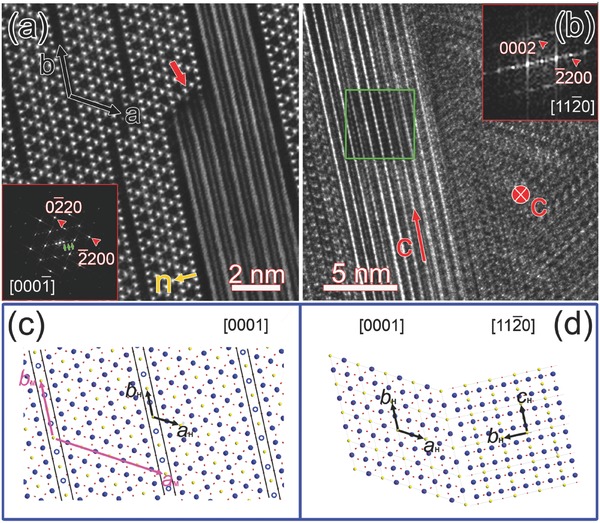
a) High‐resolution HAADF image of K*_x_*WO_3_ nanosheet including the wide‐band region. The inset is FFT of hexagonal matrix projected along ***c*** axis. b) High‐resolution TEM image of similar region including wide band indexed as $\left[11\bar{2}0\right]$. The inset in the red box is FFT of the region framed by the green box. c) Atomic model of the dark‐line superstructures constructed according to the above chemical and structural analyses on an atomic basis. The black and pink arrows indicate the hexagonal and monoclinic unit cells, respectively. d) Atomic model of the interface between the hexagonal matrix projected along [0001] and the wide band observed along $\left[11\bar{2}0\right]$.

While the dark‐line superstructures include no intergrowth tungsten bronzes, we do find regions of different structures from the HTB matrix. **Figure** **5**a is high‐resolution HAADF–STEM image of specific region in K*_x_*WO_3_ nanosheet including part of a wide band, which is penetrating through the middle of the nanosheet. The long edges of the band are parallel to the dark lines on both sides, and the interfaces between the band and hexagonal matrix are atomically clear. The band is also possible to be ended inside the nanosheet, as labeled by the red arrow, the edge of which follows the direction of specific atomic plane in the matrix. The inset of Figure 5a is fast Fourier transform (FFT) of the hexagonal matrix projected along *c* axis. Although in rare cases, there are dark‐line superstructures reappearing other than the period of 4a, their average periodicity remains 4a along *a* axis, which is reflected in the corresponding FFT as four‐fold satellite reflections indicated by green arrows.

It is quite interesting to find that while the matrix is resolved at atomic resolution, the band region could only show coarse parallel lines with the same period as the matrix planes parallel to the edges of the band. In order to figure out the structure of the band, a high‐resolution transmission electron microscopy (TEM) image of similar region is obtained as shown in Figure 5b, the contrast of which is less obvious than the high‐resolution HAADF–STEM image due to the moderate thickness of the specimen. However, the band region presents 2D crystal periodicity, which is invisible in the corresponding high‐resolution HAADF–STEM image. The inset in the green box outlines specific region filtered by the 2D masking tool of Digital Micrograph, and the inset in the red box is the corresponding FFT, which could be indexed as the diffraction along the $\left[11\bar{2}0\right]$ direction. The (−2200) reflection labeled by a red arrow is in the same direction as the (−2200) reflection of the matrix (inset of Figure 5a), which explains the same periodicity of the band as the matrix in the normal direction (labeled by the yellow arrow in Figure 5a). Moreover, the (0002) reflection of the band region indicates the *c* direction of the local nanostructure, which is perpendicular to the direction of observation in the matrix.

The loss of the crystallographic information along *c* axis in the band region in Figure 5a could be attributed to the fact that the $\left[11\bar{2}0\right]$ direction of the band is not strictly parallel to the [0001] direction of the matrix. While the matrix is projected along *c* axis, the band region is slightly tilted off the $\left[11\bar{2}0\right]$ direction with the (−2200) planes rotated somewhat around the normal axis. Since the high‐resolution HAADF–STEM imaging requires that the electron beam is strictly parallel to the atomic columns, and the high‐resolution TEM imaging is comparatively tolerant of this requirement, the orientation relationship between the band and matrix is only obtained from the high‐resolution TEM image.

Figure 5c is the atomic model of the dark‐line superstructures constructed according to the above chemical and structural analyses on an atomic basis. The average periodicity of the superstructures is 4a along *a* axis in most regions, other periodicities are also possible in rare cases. The observed dark lines take place in single atomic layers of alternating tungsten and potassium columns, as labeled by the parallel dark lines. The blue circles represent the possible distributions of W vacancies at specific sites. The intensities of the tungsten sites are variable within the layers of dark lines in some regions, which reflects variable W vacancy occupancies, as can be observed in Figure 3a indicated by pink arrows. No dark line is ever found in the neighboring layers solely composed of tungsten columns. The interface between the hexagonal matrix projected in [0001] and the wide band observed in $\left[11\bar{2}0\right]$ is modeled as presented in Figure 5d. The same lattice spacing is maintained along the interface normal, and the perfect lattice matching ensures strain fields of extremely low level and epitaxial growth at the interface.

As compared to the K*_x_*WO_3_ nanorods, the K*_x_*WO_3_ nanosheets show dark‐line superstructures with preferential orientations, and the complete structural disorder at intersecting regions of dark lines from different directions is not ever detected. The comparatively ordered superstructures within nanosheets could be related to the higher growth temperature and longer reaction time favoring the more ordered assembly of incoming constituent nanostructures. Moreover, the dark lines always bond the edges of the wide bands. The latter isolates the nanostructures into strips within which the dark‐line superstructures are inclined to be oriented along the same direction, forming large domains of ordered superstructures. Thus, it is quite possible that the extent of ordering of the dark‐line superstructures is highly dependent on the growth conditions for the nanostructures. Moreover, the existence of the isolation layers (the wide‐band regions) plays important roles in forming large domains of superstructures of the same orientation, based on which the tailoring of cation ordering is realized within single atomic layer.

For more than 40 years, formation of superlattices in hexagonal‐based potassium tungsten bronzes was attributed to K vacancies only, together with small displacements of W cations.^13^ Through simultaneous spectroscopical and structural analyses of the superstructures at atomic resolution, we find that both W and K cations participate in the formation of superlattices along ***a*** axis via periodic cation vacancy distributions within single atomic layers, and the extent of which is temperature dependent. The periodicity of the superlattices along *a* axis is 4a, which is the same as the results reported by Goodman.^13^ The small displacements of W cations within WO_6_ octahedra is reported to be ≈0.2 Å,^11^ which is out of the resolution limit of the microscope, and the oxygen atomic columns are invisible in the HAADF–STEM images. Thus, it is impossible to characterize the reported superlattices along *b* axis from the current study. Anyway, there is no conflict between our results and the former reports. A monoclinic unit cell is labeled in Figure 5c with pink arrows.

Our previous studies raised possible explanations to the observed superlattices and satellite spots in SAED patterns as forming variants in the nanosheets. Here, we do observe the proposed variants in the form of dark‐line superlattices distributed with 120° rotational symmetry. The only concern is that we do not see the orient attachment of nanorods from the cross‐sectional view of nanosheets since the crystal structures of the cross section is intact with no signs of boundaries from individual nanorods.

## 3 Conclusion

In summary, cation ordering within single atomic layer of hexagonal‐based potassium tungsten bronze nanosheets is verified both spectroscopically and structurally on an atomic basis using comprehensive TEM techniques. The observed periodic parallel dark lines are mainly attributed to small amount of W vacancies within single atomic layer. No contribution from other dopants of light elements is detected. The long periodic cation ordering within K*_x_*WO_3_ nanosheets is found to be temperature dependent, and the confined distribution of W vacancies would recover to more uniform distribution at higher temperature. The existence of the wide bands also plays important roles in forming highly ordered superstructures, since the dark‐line superstructures are inclined to be pinned along the same direction between the wide bands. Understanding the cation ordering and recovery processes from chemical and structural point of view within potassium tungsten bronze provides valuable information for tailoring cation ordering in hierarchical nanostructures, which paves the way to rational design of optoelectronic devices with controlled physical properties.

## 4 Experimental Section

*Material Growth and Sample Preparation*: The hexagonal‐based potassium tungsten bronze nanomaterials were synthesized via a vapor‐solid mechanism, by simply sonicating a clean W foil (2 × 2 × 0.025 cm) in the KOH solution (0.05 mol dm^−3^), and then heating on a hot plate under ambient conditions. While the growth temperature was maintained at 600 °C for 24 h, the morphologies of the resultant were nanosheets. The products were scratched off the substrate and sonicated in ethanol, then dropped on the holy carbon‐coated copper grid for plan‐view TEM observation. Meanwhile, some drops were left on small pieces of Si as supporter. The whole pieces were observed by electron beam, and specific nanosheet laid on the Si supporter was selected, and cut into thin slices by FIB for cross‐sectional study in TEM.

*Characterization*: The morphologies of the products were investigated by FEI SIRION field emission SEM. The cross‐sectional sample preparation was conducted using FEI Quanta 3D FEG FIB. The high‐resolution TEM images, the aberration‐corrected HAADF‐STEM images at atomic resolution and the EELS spectra were obtained using FEI Titan G^2^ 60‐300 with a probe Cs corrector operating at 300 kV and a Quantum GIF 963. The C2 aperture of 70 µm was used, and the camera length was kept at 130 mm (convergence semiangle ≈ 28.8 mrad). The chemical analysis at atomic resolution was performed using FEI Titan ChemiSTEM operating at 200 kV with the detectors super EDS energy resolution ≤ 130 eV/100 kcps. The XRD spectrum was collected using a Bruker D8 Advance X‐ray diffractometer with Cu Kα radiation (λ = 1.5418 Å). The XPS spectra were obtained using Kratos AXIS‐ULTRA‐DLD‐600W X‐ray photoelectron spectrometry. The in situ heating experiment was carried out using DENSsolution‐DH30‐4M‐JU double‐tilt heating/biasing holder.

## Supporting information

Supplementary

Supplementary
