# Supplementary material for: Atomic‐Scale Study of Cation Ordering in Potassium Tungsten Bronze Nanosheets
Source: Adv Sci (Weinh). 2017 Apr 26;4(9):1600537. doi: 10.1002/advs.201600537 (PMC5604398; doi:10.1002/advs.201600537)
Supplement: Supplementary file 1 — Supplementary [file ADVS-4-na-s001.pdf]

Copyright WILEY-VCH Verlag GmbH & Co. KGaA, 69469 Weinheim, Germany, 2017.

ADVANCED SCIENCE
Open Access

Supporting Information

for *Adv. Sci.,* DOI: 10.1002/advs.201600537

Atomic-Scale Study of Cation Ordering in Potassium Tungsten Bronze Nanosheets

Luying Li, Fan Jiang, Fanfan Tu, Shuangfeng Jia, Yihua Gao, and Jianbo Wang**

Copyright WILEY-VCH Verlag GmbH & Co. KGaA, 69469 Weinheim, Germany, 2016.

Supporting Information

Atomic-scale Study of Cation Ordering in Potassium Tungsten Bronze Nanosheets

Luying Li, Fan Jiang, Fanfan Tu, Shuangfeng Jia, Yihua Gao and Jianbo Wang**

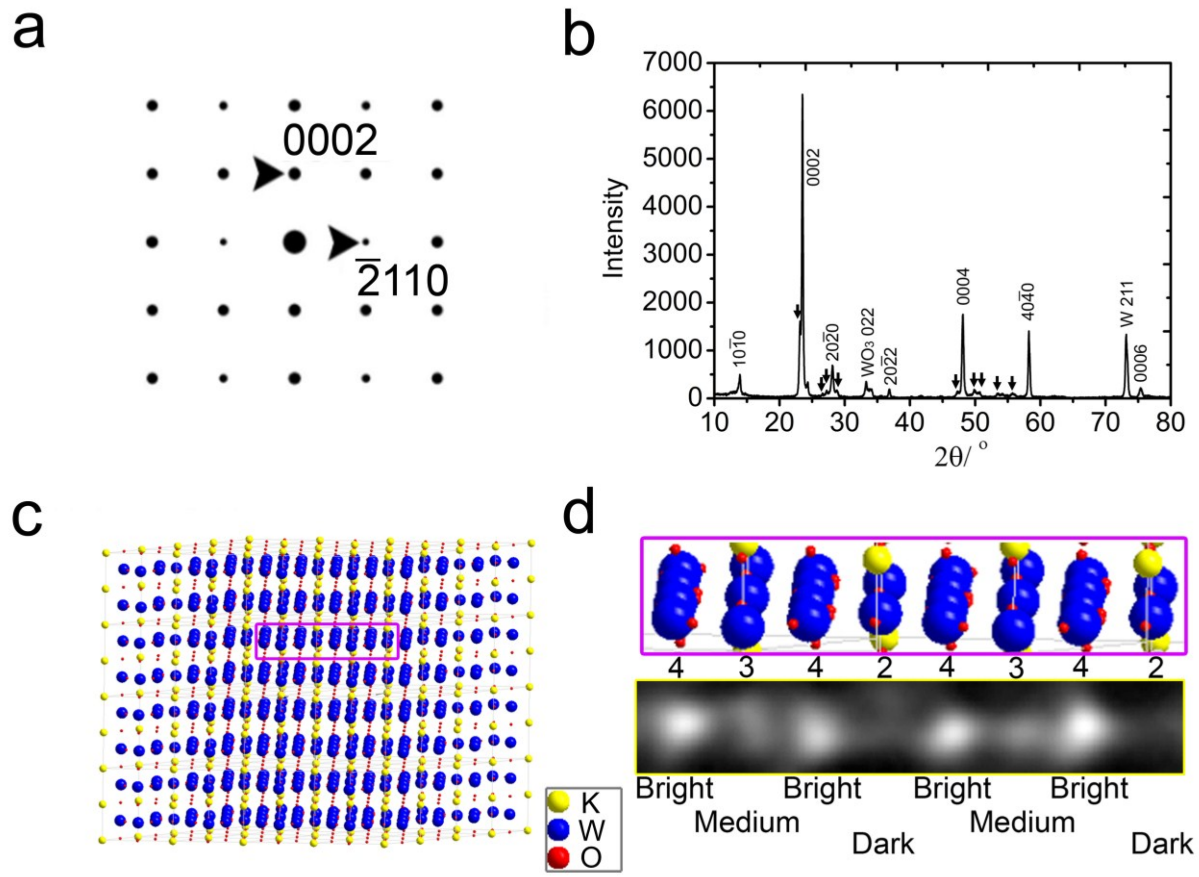

Figure S1. (a) Simulated SAED diffraction pattern of hexagonal K_xWO_3 projected along [01-10] axis. (b) The powder XRD spectrum of K_xWO_3 nanosheets. The black arrows indicate satellite superstructure peaks that could not be indexed as either hexagonal K_xWO_3 or other possible impurities. (c) 2a×2b×2c super-cell atomic model of hexagonal K_xWO_3 projected along [01-10] axis, while the projection is tilted off the zone axis a little bit to show the variable densities of atomic columns along [01-10]. The region in the pink box is magnified and shown in (d). (d) The number labeled under each atomic column indicates the number of atoms in the super cell along this projection. The image framed in the yellow box is cut out from the region in similar yellow box in Figure 1(d). The intensity of each atomic column is approximately proportional to the number of atoms in each column.

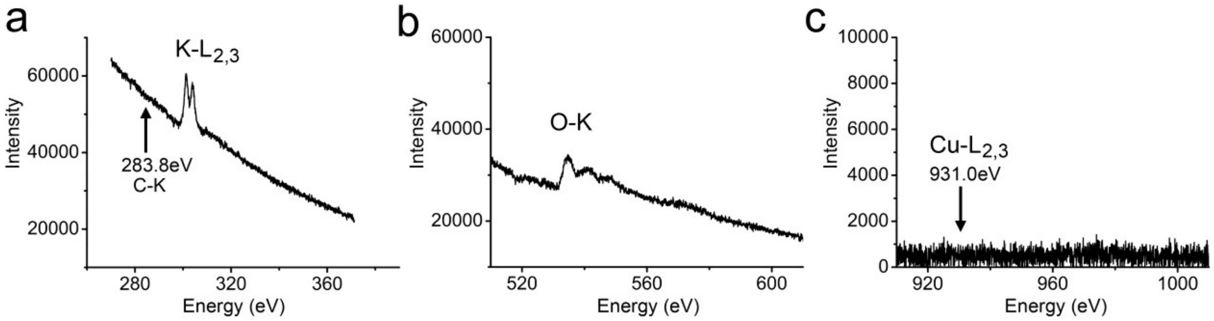

Figure S2. EELS spectra of K_xWO_3 nanosheets. The K-$L_{2,3}$ edge in (a) and the O-K edge in (b) are clearly shown, indicating the presence of K and O in the material, while the C-K edge and Cu-$L_{2,3}$ edge are absent, the supposed peak positions are labeled by black arrows in (a) and (c), respectively.

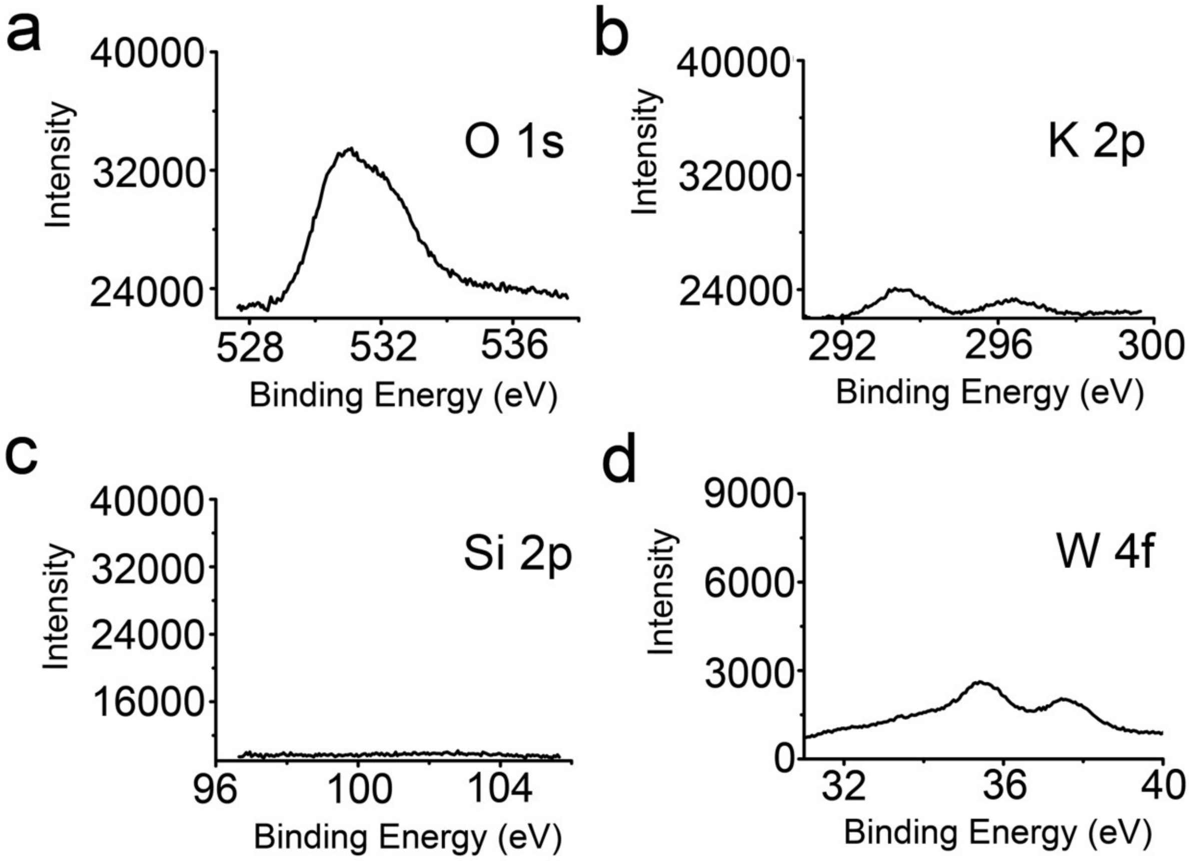

Figure S3. XPS spectra of K_xWO_3 nanosheets as grown on W foil. The O 1s and K 2p peaks are clearly shown in (a) and (b), respectively, while the Si 2p peak is absent in (c). Thus, Si is not presented in the material. (d) Signals of W 4f from K_xWO_3 nanosheets deposited on Si substrate.

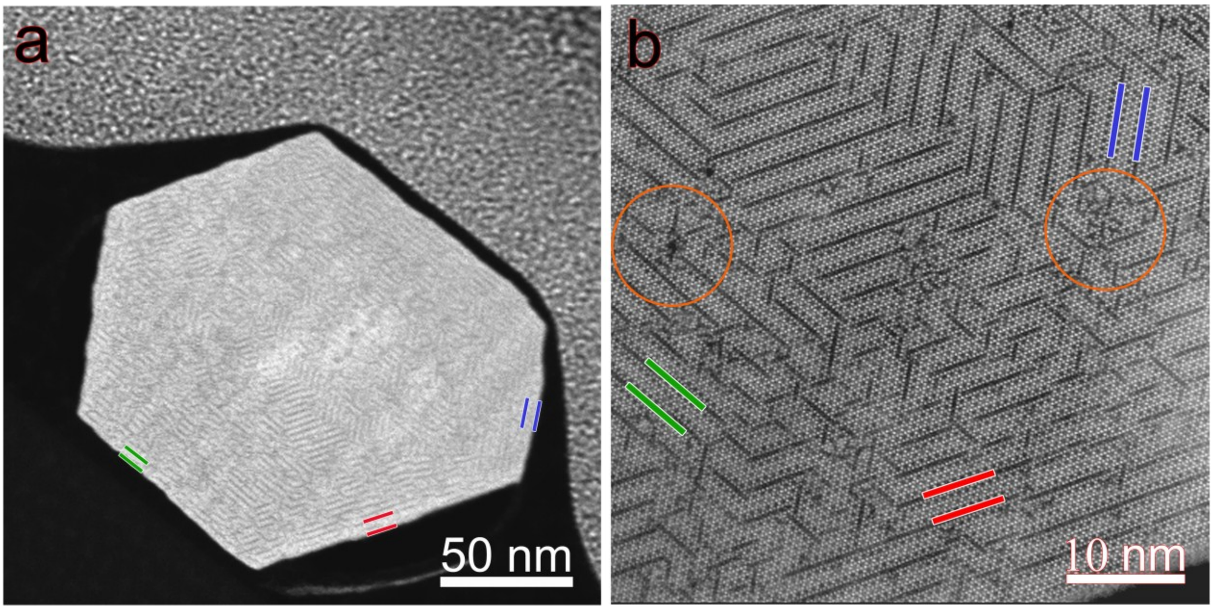

Figure S4. (a) HAADF image of cross section view of individual nanorod. The directions of the majority of the dark lines are parallel to the edges close to them, as indicated by the red, green, and blues lines. (b) High-resolution HAADF images of nanorod showing the structural features at higher magnifications. Complete disordered regions where dark lines from different directions meet together are labeled by orange circle.
